# Reorganization of the Landscape of Translated mRNAs in NSUN2-Deficient Cells and Specific Features of NSUN2 Target mRNAs

**DOI:** 10.3390/ijms23179740

**Published:** 2022-08-28

**Authors:** Olga A. Kossinova, Alexander V. Gopanenko, Elena S. Babaylova, Alexey E. Tupikin, Marsel R. Kabilov, Alexey A. Malygin, Galina G. Karpova

**Affiliations:** Institute of Chemical Biology and Fundamental Medicine, Siberian Branch of the Russian Academy of Sciences, 630090 Novosibirsk, Russia

**Keywords:** HEK293T cells, knockdown of NSUN2, PAR-CLIP, RNA-seq, polysome profiling, NSUN2 target mRNAs, NSUN2-dependent differentially expressed genes

## Abstract

The RNA cytosine C5 methyltransferase NSUN2 has a variety of RNA substrates and plays an important role in mRNA metabolism. NSUN2 binds to specific sequences enriched in exosomal mRNAs, suggesting its possible involvement in the sorting of mRNAs into exosomes. We applied the photoactivatable.4-thiouridine-enhanced cross-linking and immunoprecipitation assay involving high-throughput RNA sequencing (RNA-seq) to HEK293T cells to determine NSUN2 mRNA targets. NSUN2 cross-linking sites were found in more than one hundred relatively abundant mRNAs with a high GC content and a pronounced secondary structure. Then, utilizing RNA-seq for the total and polysome-associated mRNA from HEK293T cells with and without the knockdown of NSUN2, we identified differentially expressed genes, as well as genes with altered translational efficiency (GATEs). It turned out that the up-regulated GATE mRNAs were much shorter on average than the down-regulated ones, and their GC content was higher; moreover, they contained motifs with C residues located in GC-rich environments. Our findings reveal the specific features of mRNAs that make them potential targets for NSUN2 and expand our understanding of the role of NSUN2 in controlling translation and, possibly, in mRNA sorting into exosomes implemented through the methylation of cytosine residues.

## 1. Introduction

One of the basic cellular processes occurring in a eukaryotic cell is the post-transcriptional chemical modification of RNA bases. The data accumulated to date indicate that the methylation of cytosine residues at the C5 atoms in mammalian RNAs plays an important role in many aspects of RNA metabolism, including RNA export, RNA stability, RNA maturation, and mRNA translation [1,2]. This modification is catalyzed by enzymes of the NOL1/NOP2/SUN domain (NSUN) family, as well as the DNA methyltransferase homolog DNMT2 [3,4]. The enzyme responsible for C5 methylation in mRNAs, tRNAs, and some long noncoding RNAs (lncRNA) is the RNA cytosine-C5 methyltransferase NSUN2 [5,6]. The transcriptome-wide distribution of mRNA 5-methylcytosine (m5C) sites is now available for several human and mouse cell lines and tissues (see [7] and references therein). The patterns of mRNA m5C enrichment have turned out to be cell/tissue-dependent. In general, the accumulation of m5C sites is observed in 5′-untranslated regions (UTRs) with their prevalence near the translation initiation sites, as well as in 3′ UTRs and coding sequences (CDSs) simultaneously, while the m5C sites themselves are mainly enriched in CG motifs [7]. It has been found that m5C modifications in mRNA CDSs are associated with low translational activity in plants [8]. Similar results showing that m5C modifications in CDSs negatively affect the translation of the mRNAs have been obtained with NSUN2-knockdown human cells [7,9] and Nsun2-knockout mice [9].

The biological functions of m5C residues in mRNA metabolism remain largely unclear. Only recently, data on cellular proteins that recognize this mRNA modification have appeared. One of these proteins is the mRNA export adaptor ALYREF, proposed as an m5C mRNA reader for the regulation of nuclear mRNA export [10]. Another is the YTH domain-containing family protein 2, which has been suggested as a regulator of the levels of m5C in rRNA and mRNA [11]. Finally, Y-box-binding protein 1 (YB-1), a key regulator of gene expression [12,13,14] and a crucial player in the sorting of ncRNA into exosomes [15,16], has been reported to bind to methylated mRNA via the direct interaction of its cold shock domain with the m5C site; thereby the protein contributes to mRNA stabilization [17].

We have previously demonstrated that NSUN2, along with YB-1, can be pulled down from a cytoplasmic extract of human embryonic kidney 293 (HEK293) cells using a short synthetic imperfect RNA hairpin containing the linear octanucleotide sequence CAGUGAGC (motif 2) [18]. Similar RNA hairpins comprising sequences ACCAGCCU (motif 1) or UAAUCCCA (motif 3) were specific to YB-1 rather than NSUN2 [18]. At that, both proteins have been found in the exosomal cargo from HEK293 cells [18]. Considering that all three motifs are often present in exosomal RNAs [19], we have suggested that YB-1 and NSUN2 are involved in the sorting of mRNAs into exosomes [18]. Later, we extended motifs 1–3 to the corresponding degenerate consensus sequences 1–3, which are widely abundant in the 3′ UTRs of human mRNAs [20,21] interacting with YB-1 [21]. Given recent data indicating an increase in the stability of m5C-modified mRNAs due to interaction with YB-1 [17,22], it can now be assumed that before mRNAs with the consensus sequences 1–3 are recognized by YB-1, they undergo C5-methylation.

In connection with the foregoing, it was of interest to determine the specific features of NSUN2 binding regions in cellular mRNAs, substrates for NSUN2, and also find out whether the translation efficiencies of the mRNAs increase in response to a decrease in the enzyme level. To reveal NSUN2 target mRNAs, we applied the photoactivatable ribonucleoside-enhanced cross-linking and immunoprecipitation (PAR-CLIP) method to HEK293T cells grown in the presence of 4-thiouridine (s^4^U). By analyzing samples of total mRNA and polysome-associated mRNAs from cells with or without NSUN2 knockdown using high-throughput RNA sequencing (RNA-seq), we identified differentially expressed genes (DEGs) at the transcriptional and translational levels, as well as genes with altered translational efficiencies (GATEs). Thus, mRNAs cross-linked to NSUN2 were defined, and the impact of NSUN2 deficiency on the cellular transcriptome and translatome was revealed.

## 2. Results

### 2.1. NSUN2 In-Cell Cross-Linking

**Figure 1:**
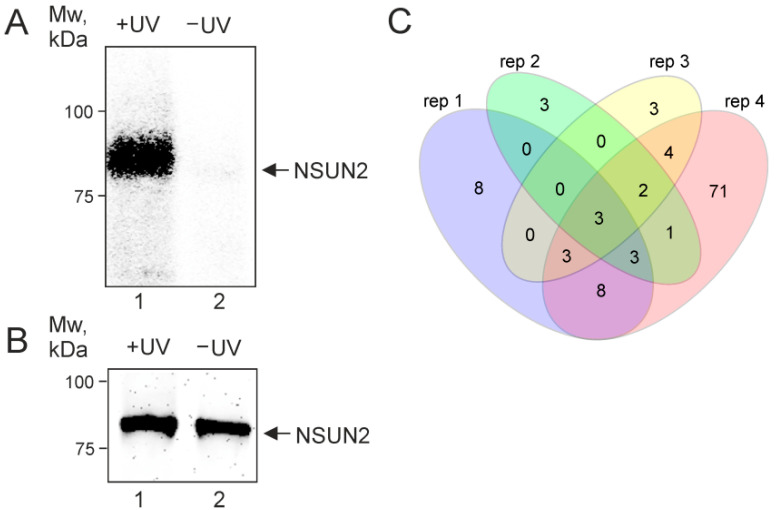
In-cell s^4^U-dependent cross-linking of RNAs to NSUN2 induced by mild UV irradiation. (**A**) The autoradiograph of the nitrocellulose membrane showing SDS-PAGE-purified NSUN2-RNA cross-links with protein molecular weight marker positions indicated on the left. (**B**) Western blot analysis of the same membrane with the use of anti-NSUN2 antibodies showing immunoprecipitated NSUN2. Arrows indicate the position of NSUN2. The electrophoretic mobility of the labeled cross-linked protein does not noticeably differ from that of the unmodified protein because relatively short cross-linked RNA fragments do not significantly contribute to the mobility of large proteins (see [26]). (**C**) Venn diagram showing the overlap of the sets of genes encoding mRNAs cross-linked to NSUN2 found in four biological replicates.

The interaction of endogenous NSUN2 with mRNAs was studied using the PAR-CLIP method [23,24,25] with HEK293T cells grown in the presence of s^4^U. After irradiation of the cells with mild UV light, NSUN2 was immunoprecipitated from the RNase T1-treated cell lysate with the use of specific antibodies. RNA fragments cross-linked to NSUN2 were 5′-^32^P-labeled and purified by SDS-PAGE, followed by transfer onto a nitrocellulose membrane, which prevented their contamination with free RNA. The autoradiograph of the membrane exhibited a strong signal for the sample from irradiated cells, which was not observed for that from non-irradiated ones (Figure 1A and B, lanes 1 and 2, respectively). This indicated the generation of s^4^U-dependent NSUN2-RNA cross-links in UV-irradiated cells and, accordingly, the presence of RNA fragments cross-linked to the protein in lane 1 (Figure 1A). The RNA fragments recovered from the excised and proteinase K-treated membrane piece corresponding to the RNA-NSUN2 cross-links were converted into a cDNA library that was subjected to next-generation sequencing (NGS). In total, the experiment was carried out in four biological replicates.

### 2.2. NSUN2-Cross-Linked RNAs

NGS data analysis revealed regions of the human genome covered by about two thousand peaks containing sequencing reads with characteristic T/C transitions (Table S1) that occurred because the reverse transcriptase used in the library preparation recognizes cross-linked s^4^U residues as cytidine ones [23]. Therefore, the locations of peaks on the genome map correspond to RNA fragments cross-linked to NSUN2, and the positions of the T/C transitions in the reads indicate those of the respective cross-linking sites in these RNA fragments. Approximately half of the peaks are attributed to protein-coding genes, and another part refers to the genes of different RNA biotypes (mainly lncRNA, rRNA, snRNA, misc_RNA, and tRNA), as well as to overlapping (multiple) genes (Table S1). This is consistent with earlier reports that NSUN2 has diverse RNA substrates, including rRNAs, tRNAs, mRNAs, and vtRNAs (see [3] for a review).

**Figure 2:**
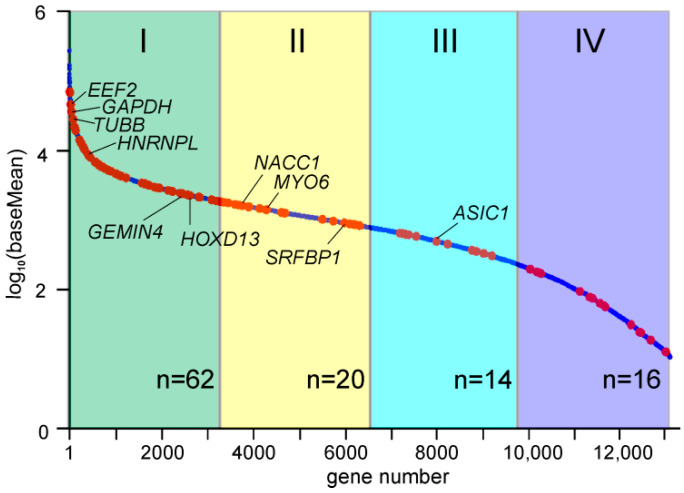
The abundance of NSUN2-cross-linked mRNAs. The graph shows the order of genes relative to their mRNA abundance, defined as the baseMean values obtained in an RNA-seq experiment with total mRNA from HEK293T cells (the line of blue dots). Red dots correspond to the genes whose mRNAs were cross-linked to NSUN2. The quartiles of genes are colored and numbered with Roman numerals. The number of red dots in each quartile (n) is shown at the bottom. Representative genes are designated.

**Figure 3:**
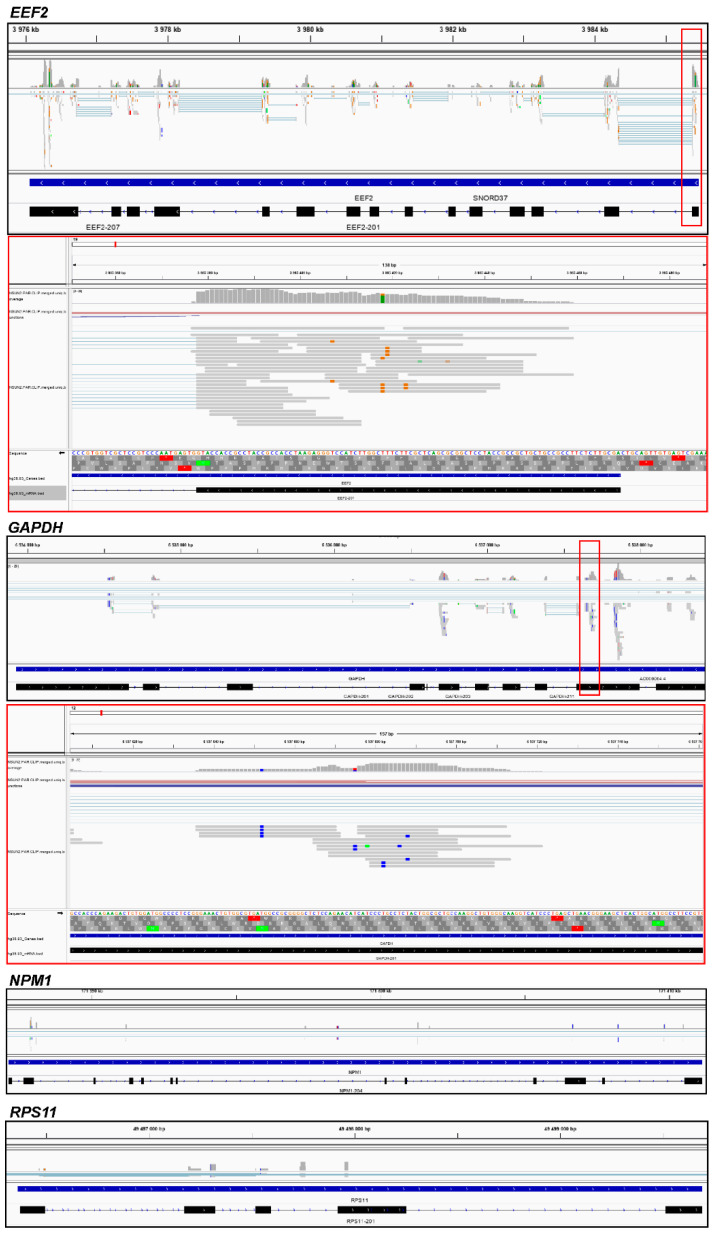
The IGV genome browser view of regions of the human genome corresponding to the representative genes *EEF2* and *GAPDH* with the mapped sequencing reads obtained by the NGS of the RNA fragments cross-linked to NSUN2. Zoomed regions of the browser view are shown in red boxes. The positions of the T/C transitions in the reads are visible as vertically repeating blue (or brown for the *EEF2* gene) dashes above the T letters in the sequence line. Views of the *NPM1* and *RPS11* gene regions showing no reads are presented for comparison as a control for the specificity of pull-down of the NSUN2-cross-linked mRNA fragments. Additional samples are presented in Figure S1.

Since our task was to establish NSUN2 targets among mRNAs, we next focused on the protein-coding genes. The 235 peaks with reads containing T/C transitions in the exon regions of 121 protein-coding genes were found in the reads pooled from four biological replicates (Table S1), and these genes overlapped fairly well in the replicates (Figure 1C). Most of these peaks (192) were in the coding parts of these genes, although a small part of them fell into the gene regions corresponding to the 3′ and 5′ UTRs of their mRNAs (Table S1). The set of the nuclear coding genes was mainly represented by highly and moderately expressed genes with various biological functions (e.g., *EEF2*, *GAPDH*, *TUBB*, *RPL12*, *HSPA1B*, *SFPQ*, *IRS4*, *GEMIN4*, *HOXD13*, and *NACC1*), as well as histone genes (e.g., *H1–2*, *H1–4*, *H3C2*, and *H4C4*) (Figure 2 and Figure 3 and Table S1). Judging by the small number of mRNAs found to be cross-linked to NSUN2, it can be assumed that the protein does not have preferred targets among mRNAs and more frequently binds to those that are more abundant (Figure 2). At the same time, it should also be taken into account that we could only observe NSUN2 cross-links with mRNA regions in which the s^4^U residue was in a suitable position near the protein binding site containing the target for methylation, a cytidine one. Moreover, the regions favored for NSUN2 binding should definitely not have many s^4^U residues available for cross-linking since the median GC content value in sequences covered by the read peaks is approximately 60% (Table S1), implying that they are GC-rich (for comparison, the total GC content in human mRNA is 48.8% [27]). It is also noteworthy that the mRNA regions corresponding to the peaks with the T/C transition-containing reads have a rather pronounced secondary structure (~44% of all nucleotides in these regions form complementary pairs) (Table S1).

We compared the set of NSUN2 target mRNAs found by PAR-CLIP in the current study with the set of 312 mRNAs that have previously been identified as contacting this protein using the methylation individual-nucleotide-resolution cross-linking and immunoprecipitation (miCLIP) method based on the spontaneous cross-linking of the NSUN2 C271A mutant to an RNA substrate [28]. These sets overlapped by only 11 mRNAs, which is most likely due to differences between the cross-linking mechanisms in the above CLIP methods. The total number of NSUN2-cross-linked mRNAs detected by PAR-CLIP and miCLIP is not as large as might be expected, indicating the poor availability of potential methylation sites in cellular mRNAs for NSUN2, probably because of the involvement of most of them in translation. It is noteworthy that a fairly significant part of mRNAs found to be cross-linked to NSUN2 using PAR-CLIP (23 out of 121) is also present in the set of mRNAs identified earlier by the same method as those cross-linked to YB-1, the protein that provides packaging for untranslated mRNAs [21]. A total of 6 of these 23 mRNAs contain the degenerate consensus sequence with motif 2 in their 3′ UTRs, often found in exosomal RNAs, which has previously been noted as being specifically recognized by NSUN2 (see the Introduction) [20,21], although the NSUN2 cross-linking sites in these mRNAs lie far beyond the consensus sequences. Certainly, the methylation of mRNAs by NSUN2 regulates their fate, and for some mRNAs, this modification possibly promotes their packaging with the participation of YB-1 and subsequent transfer into exosomes.

### 2.3. Knockdown of NSUN2

**Figure 4:**
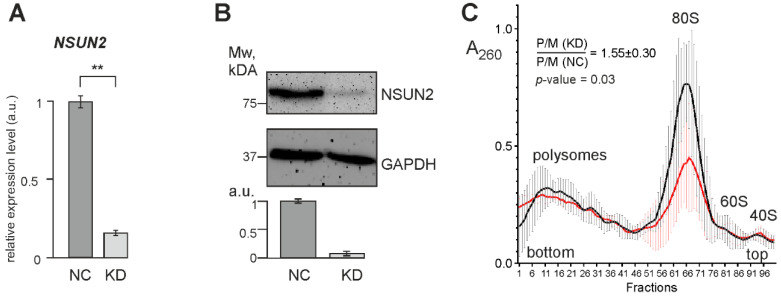
The characterization of HEK293T cells with NSUN2 knockdown. (**A**) The RT-qPCR analysis of the NSUN2 mRNA level in cells treated with NSUN2 mRNA-specific siRNAs (KD) and cells treated with control non-targeting siRNA (NC), presented as the mean of arbitrary units (a.u.) from 4 replicates ± SEM (** *p* < 0.002, calculated using Mann–Whitney test). (**B**) The Western blot analysis of the contents of NSUN2 and GAPDH (as a reference) in cells transfected with siRNAs specific to NSUN2 mRNA (KD) and cells treated with non-targeting siRNAs (NC). The bar chart shows the Western blot data as the mean of a.u. ± SEM (*p* < 0.005, calculated using Mann–Whitney test). (**C**) Polysome profiles obtained from the lysates of HEK293T cells transfected with either NSUN2 mRNA-specific siRNAs (red line) or non-targeting siRNAs (black line). The mean profiles are shown as curves drawn through 100 points with error bars, which correspond to the average absorbance at 260 nm ± SD in 4 replicates. Peaks corresponding to polysomes, 80S monosomes, and 60S and 40S ribosomal subunits are marked. The mean ratio P/M(KD):P/M(NC) with the SD determined from four biological replicates and its significance (as the *p* value) is shown. Polysome profiles obtained with cell lysates from each biological replicate are presented in Figure S2.

To identify genes whose expression changes with a decrease in the efficiency of NSUN2-catalyzed RNA methylation, we knocked down this protein via RNA interference. To achieve this, HEK293T cells were treated with specific siRNAs designed against two regions in the CDS of NSUN2 mRNA (Table S2), whereas non-targeting siRNAs were used in control experiments. The quantification of the knockdown efficiency, verified by RT-qPCR (Figure 4A) and Western blotting (Figure 4B), showed that the levels of NSUN2 mRNA and the protein were reduced by a factor of 6–7 and 10, respectively. Lysates from cells with reduced NSUN2 levels and from control cells were subjected to centrifugation in a sucrose density gradient to generate polysome profiles. The resulting profiles showed a noticeable difference in the polysome to monosome ratio (P/M) between these cell samples (Figure 4C and Figure S2), meaning that translation in polysomes was stimulated in cells with reduced NSUN2 levels.

### 2.4. Differentially Expressed Genes of Cells with and without NSUN2 Knockdown

**Figure 5:**
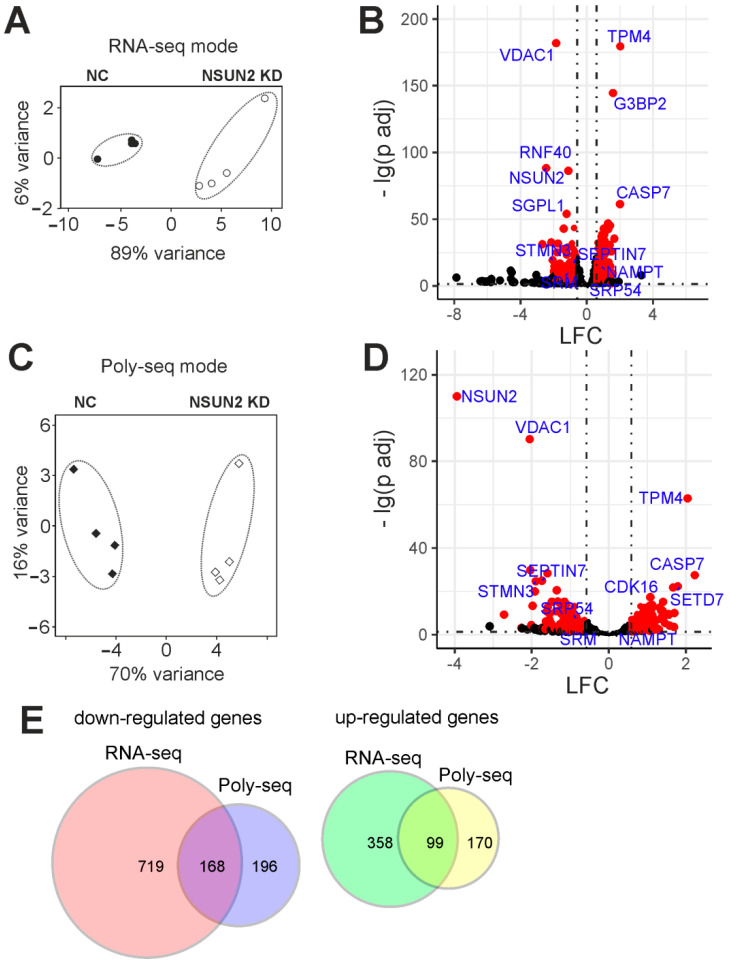
The summary of the results of RNA-seq and polysome profiling followed by RNA-seq (Poly-seq) modes with NSUN2-deficient HEK293T cells. (**A**) The principal components analysis (PCA) plot for the RNA-seq data (4 biological replicates). (**B**) A volcano plot showing the results of the differential expression analysis with DESeq2 for the RNA-seq data. Each dot corresponds to a single gene plotted according to its shrunken Log2FoldChange (LFC) and the negative logarithm of the adjusted *p* value (*p* adj). Genes sorted according to the criteria specified in Section 4 (DEGs) are shown as red dots. Cut-off parameters are shown as dotted lines (*p* adj < 0.05, |LFC| > 0.585, which corresponds to a 50% change in the gene expression). (**C**) PCA plot for Poly-seq data (4 biological replicates). (**D**) A volcano plot for the results of DESeq2 analysis with Poly-seq data. Cut-off parameters were the same as for RNA-seq data. (**E**) Venn diagrams representing the overlap of the down-regulated and up-regulated gene sets found using RNA-seq and Poly-seq.

To reveal differences in mRNA compositions in the total RNA and total polysomal RNA patterns between NSUN2-knockdown and control cells, the RNA-seq analyses of the respective RNA samples were performed. The evaluation of the RNA-seq data by the principal component analysis (PCA) showed a high degree of clustering between biological replicates in both sequencing modes: RNA-seq (Figure 5A) and polysome profiling followed by RNA-seq (Figure 5C). This implied that the data obtained were of high quality and suitable for further downstream processing. First, the raw sequencing reads were filtered and quality-checked, then mapped to the reference human genome, where they predominantly fell into the regions corresponding to protein-coding transcripts (Tables S3 and S4). The subsequent differential expression analysis with RNA-seq and polysome profiling followed by RNA-seq data revealed sets of genes whose expression was altered at the transcriptional and translational levels, respectively, in NSUN2-deficient cells compared to those treated with non-targeting siRNAs (Table S2). From these sets, statistically significant transcriptionally and translationally differentially expressed genes (i.e., those whose mRNA contents were altered in total mRNA and/or polysome-associated mRNA samples from the cells with the NSUN2 deficiency, abbreviated as tDEGs and pDEGs, respectively), were selected using the defined cut-off parameters. The adjusted *p* value (*p*.adj) was taken below 0.05 to sort out statistically significant genes between all replicates, and the minimal absolute shrunken Log2FoldChange (LFC) value was limited to 0.585 to only consider genes with more than 1.5-fold changes in expression levels (Figure 5B,D). Applying these cut-offs, we identified a set of tDEGs consisting of 887 down-regulated and 457 up-regulated genes (Table S3), as well as a set of pDEGs comprising 364 and 269 such genes (Table S4), respectively. The sets of down-regulated tDEGs and pDEGs, as well as the sets of up-regulated tDEGs and pDEGs, only partially overlapped (Figure 5E).

**Figure 6:**
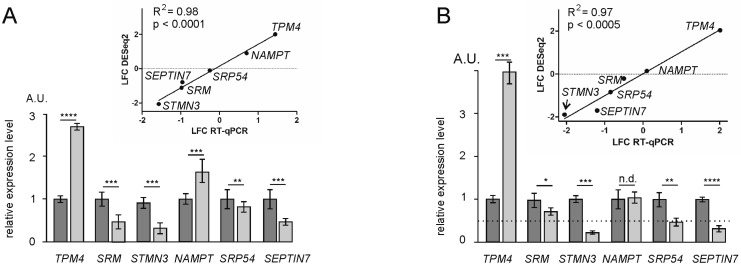
The validation of differential gene expression analysis results by RT-qPCR. Data are presented as the mean of arbitrary units (A.U.) from three or more replicates ± SEM (* *p* < 0.05, ** *p* < 0.01, *** *p* < 0.001, **** *p* < 0.0001 (Mann–Whitney test), n.d., no difference); light and dark columns represent data for knocked-down cells of NSUN2 and cells treated with non-targeting siRNAs, respectively. Relative levels of mRNAs for the selected tDEGs were determined for samples of total mRNA (**A**) and of mRNA from polysomes (**B**). The correlation between RT-qPCR and RNA-seq/Poly-seq data is shown above the columns.

The results of the differential expression of genes and their translational efficiencies analyses obtained with NSUN2-deficient HEK293T cells were validated by RT-qPCR. To accomplish this, six tDEGs, namely *SRM*, *STMN3*, *TPM4*, *SRP54*, *NAMPT*, and *SEPTIN7*, were selected. The values of changes in the expression of these genes at the transcriptional level and in their translation efficiencies, estimated using RT-qPCR, highly correlated with those obtained from analyzes with the NGS data (Figure 6).

To reveal the features of the identified DEGs, we performed Gene Ontology (GO) enrichment analysis for the up-regulated and down-regulated DEG sets. No enrichment for the up-regulated tDEGs and pDEGs was found in the biological process and molecular function categories. However, both sets exhibited weak enrichment in the cellular component category for the genes whose products are associated with lipid membranes and membrane-bound organelles, such as, in particular, the endoplasmic reticulum (ER), nuclear lumen, and centriole (Table S5). In contrast, the set of down-regulated pDEGs showed no enrichment in all three categories of the GO database. As for the set of down-regulated tDEGs, it displayed enrichment for the GO biological process terms linked to the ER and the regulation of neuron activity (Table S5). In the GO cellular component category, these genes turned out to be related to very specialized cellular components, such as the laminin-11 complex, synaptic cleft, synaptic vesicle membrane, neuron projection terminus, and distal axon, associated with the transmission of nerve impulse (Table S5). Thus, the results of the GO enrichment analysis for the up-regulated DEGs suggest that the reduced level of NSUN2 leads to an increase in the cellular contents of mRNAs for many membrane proteins and the activation of their translation. Since membrane proteins are synthesized by ribosomes bound to the ER, one can say that the knockdown of NSUN2 results in enhanced translation on it, which is apparently responsible for the observed increase in the share of polysomes in NSUN2-deficient cells (see Figure 4C). At the same time, the GO enrichment in cellular components linked to the transmission of nerve impulses found for the down-regulated tDEGs can indicate an important role of NSUN2 in maintaining the work of neurons.

### 2.5. Genes with Differential Translation Efficiencies for Cells with and without NSUN2 Knockdown

The routine analysis of tDEGs and pDEGs showed that the differential expression of genes at the level of translation was not derived from that at the level of transcription (see Figure 5E and Tables S3 and S4). Many down-regulated and up-regulated tDEGs were not translationally regulated, and conversely, a number of pDEGs were not transcriptionally regulated. Therefore, in order to learn how NSUN2 deficiency affects the efficiency of the translation of specific mRNAs, we performed a differential analysis using the RNA-seq data and the polysome profiling followed by RNA-seq data simultaneously and found genes with altered translation efficiency (GATEs) (Table S6). Using the cut-off parameters mentioned above, we identified 323 up-regulated and 273 down-regulated GATEs. The GO enrichment analysis for the up-regulated GATEs showed that they are mainly involved in the biological processes associated with mitochondrion organization, cellular localization, phosphorylation, and protein metabolism (Table S5). Down-regulated GATEs were mainly enriched in genes implicated in biological processes that regulate the cell cycle and in those such as cell division, the establishment of chromosome localization, mitotic spindle organization, and mRNA metabolism (Table S5). It is noteworthy that *NSUN2* itself appeared in the set of down-regulated GATEs; the level of its mRNA in the pool of polysome-associated mRNAs (Table S4) decreased much more significantly than in the pool of total cellular mRNAs (Table S3).

**Figure 7:**
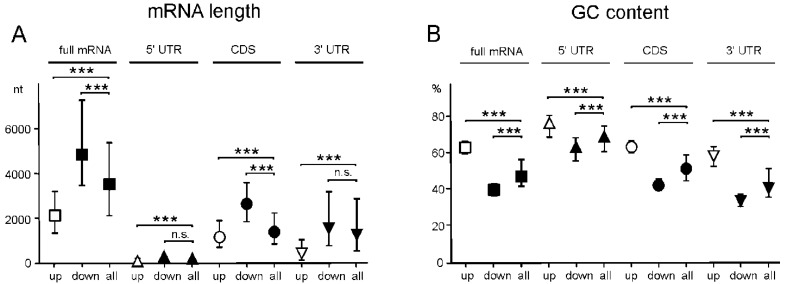
Characteristic features of mRNAs of up-regulated and down-regulated genes with altered translational efficiencies (GATEs) found for NSUN2-deficient HEK293T cells. Calculated median values with interquartile ranges of length (**A**) and GC content (**B**) for full-length mRNAs and their coding and untranslated regions corresponding to up-regulated (up) and down-regulated (down) GATEs and for all mRNAs of HEK293T cells (all) (*** *p* < 0.001, n.s. not significant (Mann–Whitney test)).

To determine whether mRNAs of GATEs have characteristic features, we calculated the median values of the length and GC content of these mRNAs, as well as of their functional parts. It turned out that, on average, the length of mRNAs of up-regulated GATEs was much shorter than that of human mRNAs, while the length of mRNAs of down-regulated GATEs was significantly longer (Figure 7A). It is noteworthy that the same was also true for individual functional parts of the GATE mRNAs (5′ UTRs, CDSs, and 3′ UTRs) (Figure 7A). As for the GC content, on the contrary, it was higher on average for mRNAs of up-regulated GATEs and lower for mRNAs of down-regulated GATEs than for all mRNAs, and such a correlation was observed not only for full-length mRNAs but also for their functional parts (Figure 7B). All this indicates that the knockdown of NSUN2 results in the enhanced translation of shorter mRNAs with higher GC content.

**Figure 8:**
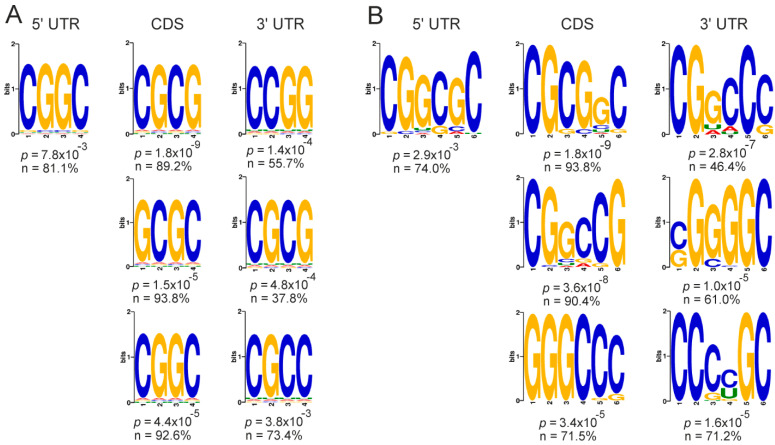
The most frequently occurring motifs found in mRNAs of up-regulated GATEs and presented as 4-mers (**A**) and 6-mers (**B**) in their 5’ UTRs, CDSs, and 3’ UTRs with the indication of *p*-values and percentages of mRNA sequences containing the particular motif in certain functional part. For CDSs and 3′ UTRs, the 3 most representative motifs are given.

Next, we searched for specific 4- and 6-mer motifs in the mRNAs of the GATEs. A high occurrence of the motifs with G and C repeats in different variants was found in all functional parts of the up-regulated GATE mRNAs (Figure 8), while no high-probability motifs were revealed in the down-regulated ones (data not shown). This implies that the cellular deficiency of NSUN2 leads to an increased translation of mRNAs, in which C residues, regardless of where they are located in the mRNAs, are often found in close proximity to G residues.

## 3. Discussion

In this work, using NGS-based approaches, we revealed the main mRNA targets of the RNA cytosine C5-methyltransferase NSUN2 and found out how the compositions of mRNA pools presented in the transcriptome and translatome of HEK293T cells change with a decrease in the level of this enzyme. Utilizing the PAR-CLIP method with s^4^U as a photoactivatable nucleoside analog, we performed the intracellular cross-linking of endogenous NSUN2 with its RNA targets and identified more than a hundred mRNAs among RNAs of different biotypes cross-linked to this protein. The levels of these mRNAs in cells are relatively high, they encode proteins with diverse biological functions, and their regions with NSUN2 cross-linking sites have a high GC content. With a 10-fold decrease in the level of NSUN2 achieved using RNA interference, we observed an increase in the level of polysomes, which indicates the stimulation of translation caused by the NSUN2 deficiency. The application of RNA-seq to samples of total RNA and RNA isolated from the polysome fraction from NSUN2-knockdown cells and to similar samples from cells treated with non-targeting siRNAs allowed us to identify hundreds of tDEGs and pDEGs. Moreover, we showed that differential gene expression at the translational level is independent of that at the transcriptional level, which points to changes in the efficiencies of translation of certain mRNAs. Indeed, via joint analysis of the sets of identified tDEGs and pDEGs, we revealed 323 up-regulated and 273 down-regulated GATEs, i.e., genes with increased and decreased translational efficiencies, respectively. Characteristic features of the up-regulated GATE mRNAs are that, compared to the down-regulated ones, they are shorter on average and have a higher GC content. In addition, these mRNAs often contain motifs in which C residues are in GC-rich environments. The obtained data provide new information on mRNA targets for NSUN2 and the regulation of cellular translation through the specific C5-methylation of cytosine residues in mRNAs by this enzyme.

The presence of mRNAs encoded by highly expressed genes involved in various cellular processes among those cross-linked to NSUN2 may indicate that NSUN2 has no selectivity for any particular cellular mRNAs. Like other researchers [10,28], we failed to identify any clear linear motif in mRNAs, which could be recognized by NSUN2, so we made an assumption that either such a motif is very degenerate or the protein recognizes some 3D structural element(s) in mRNAs. The latter is supported by our finding that mRNA regions containing NSUN2 cross-linking sites have a more pronounced secondary structure (~44% nucleotide residues form complementary pairs) than mammalian mRNAs on average (~30–35% nucleotide residues can be paired [29]). It appears that the appropriate GC-rich environments of the target cytosine residues in these mRNA regions are sufficient for their NSUN2-catalyzed methylation.

As mentioned in the Introduction, of short RNAs with motifs 1–3 often found in exosomal mRNAs, only RNA with a degenerate consensus sequence containing motif 2 was able to bind to NSUN2 [18]. Among the degenerate consensus sequences containing motifs 1–3 [21], only the sequence with motif 2 in an extended hairpin has C residues in a GC-rich environment, which is consistent with the concept of the NSUN2 binding site on mRNA obtained in this study. However, although the set of mRNAs cross-linked to NSUN2 included those containing consensus sequences 2 in their 3′ UTRs, we did not find NSUN2 cross-linking sites within these sequences. Moreover, the majority of the cross-linking sites were found in the CDSs, although there are much fewer consensus sequences 2 in these parts of mRNAs than in 3′ UTRs [18,21]. Under in cellulo conditions, the 3′ UTRs of mRNAs are usually occupied by a large number of RNA-binding proteins, which, certainly, largely shield them from the action of NSUN2, while ribosome-translated CDSs are more accessible for the m5C modification. One of the proteins that bind to 3′ UTRs could be YB-1, which, as already noted, binds to m5C sites [17,22] and is most likely involved in mRNA sorting into exosomes [16]. In this line, one can assume that the recognition of the consensus sequence 2 [18,21] in the mRNA 3′ UTRs by NSUN2, followed by the methylation of cytosine residues in regions with appropriate GC environments, triggers their binding to YB-1, promoting multimerization of the latter at the 3′ UTRs. Therefore, the methylation of mRNAs containing consensus sequence 2 in the 3’ UTRs with NSUN2 may be an initial stage of mRNA sorting into exosomes, which is turned on by cells, if necessary, via special signaling mechanisms.

To date, there is sufficient evidence of a negative correlation between the levels of m5C modifications in the CDSs of mRNAs and their translation efficiencies [7,8,9], which in turn means that NSUN2 can regulate protein synthesis by reducing the levels of translation of its target mRNAs through their methylation. Therefore, a decrease in the level of NSUN2 should enhance the translation of mRNAs that are substrates for NSUN2. Since among these mRNAs, there are a lot of those whose content in cells is quite high, the overall level of translation should also increase upon NSUN2 deficiency, which is what we observe in the current study (Figure 4C). Cytosine residues in GC-rich contexts in the mRNAs of up-regulated GATEs found for NSUN2-knockdown cells could be potential methylation sites, which is also consistent with our observation that mRNA sequences cross-linked to NSUN2 were enriched in GC content.

It has previously been shown that NSUN2 knockout in mice, on the contrary, reduces the efficiency of protein synthesis, which is apparently caused by the loss of NSUN2-dependent tRNA methylation [30]. With NSUN2 knockdown, the effect of reducing tRNA modifications is most likely not very significant since the half-life of tRNA in cells is 36–60 h [31], which exceeds the time of culturing cells with siRNAs in our work, and therefore there should not be a noticeable depletion of cells by methylated tRNAs. Notably, the translation of the NSUN2 mRNA itself is also regulated by methylation, as evidenced by the presence of the *NSUN2* gene among down-regulated GATEs. The translational efficiency of this gene was reduced 2.3 times in cells with NSUN2 mRNA knockdown, with a 6–7-fold decrease in the level of the latter and more than a 10-fold decrease in the protein level.

**Figure 9:**
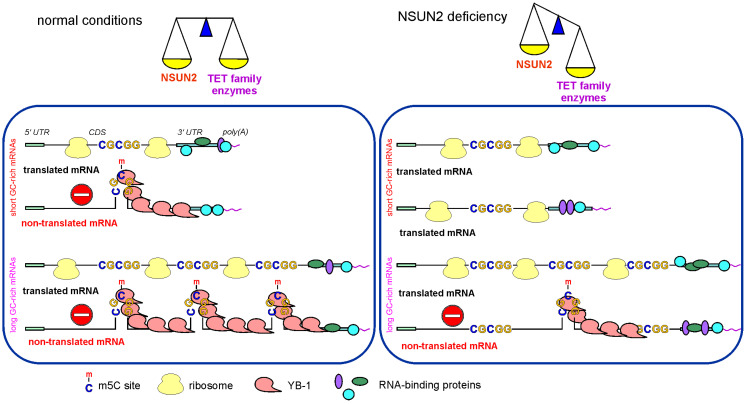
Scheme showing a possible molecular mechanism of the regulation of mRNA translation with the involvement of NSUN2. Left panel, translated mRNAs under conditions of normal NSUN2 activity; right panel, translated mRNAs under NSUN2 knockdown conditions.

Why does a decrease in the cellular level of NSUN2 lead to specific changes in the efficiency of mRNA translation? Why is the translation of shorter mRNAs enhanced while the translation of longer mRNAs decreases, and why do up-regulated GATE mRNAs contain GC-rich motifs that closely resemble NSUN2-methylation sites, while down-regulated GATE mRNAs lack such motifs? It is likely that in NSUN2 knockdown, the levels and activities of demethylases of the TET family, enzymes that remove methyl groups from m5C [3], remain unchanged, which should shift the balance between mRNA methylation and demethylation towards demethylation. This means an increase in the number of demethylated mRNAs in the set of NSUN2 target mRNAs. At the same time, the share of shorter mRNAs among demethylated ones should be higher since they have, on average, fewer methylation sites per molecule than longer mRNAs. Thus, with NSUN2 deficiency, one should expect an increment in the pool of translated mRNAs, along with some increase in translation efficiency (Figure 9), which is what we observe in cells with NSUN2 knockdown (Figure 4C). In addition, in this case, if the translational potential of the cell, determined by the number of ribosomes, remains unchanged, the density of ribosomes per mRNA should decrease. This should lead to larger accessibility for RNases and a greater propensity for degradation of longer mRNAs, and the pool of translated mRNAs should be redistributed towards increasing the efficiency of translation of shorter mRNAs. The same is roughly observed in this study (Figure 7).

At the same time, one cannot exclude a possibility of an indirect involvement of NSUN2 in the regulation of mRNA translation and RNA metabolism in general. Since NSUN2 deficiency leads to an increase/decrease in the translation of individual mRNAs, it is reasonable to expect an enhancement/reduction in the levels of the proteins encoded by these mRNAs, which, in turn, might also affect translation efficiency and gene expression. For example, the *DUS3L* and *MSI1* genes encoding the respective RNA-binding proteins can be noticed in the up-regulated GATEs set. The first of these proteins is able to mediate the formation of dihydrouridine in some mRNAs, thereby regulating their translation [32], and the second is an RNA-binding protein that contains RNA-recognition motifs type 1 and 2 and regulates the expression of certain mRNAs [33]. That is, we can expect a change in the translation efficiency of target mRNAs of these proteins in NSUN2-deficient cells. On the other hand, among the down-regulated GATEs, one can observe the genes of such RNA-binding proteins as G3BP1, DDX1, HNRNPH2, and PRPF40A. The first two proteins are RNA helicases, which play an essential role in the packing of mRNAs into stress granules (SG) [34,35] which occurred with the participation of YB-1, and therefore their deficiency should reduce the efficiency of mRNA transfer to SG. The last two proteins are important players in spliceosome formation [36], and their deficiency is expected to affect the rate of splicing and formation of mature mRNAs. All this indicates rather complex and diverse pathways for the involvement of NSUN2 in the regulation of mRNA translation and biogenesis.

Thus, the identification of GATEs in NSUN2-deficient cells, together with the found features of their mRNAs, shed light on the mechanism of regulation of the cellular translatome by NSUN2. The determination of NSUN2 binding sites on mRNAs expands the available information on the principles of recognition and methylation of mRNAs by this enzyme.

## 4. Materials and Methods

### 4.1. Materials

All oligodeoxyribonucleotides used in this study (Table S7) were synthesized in the Laboratory of Biomedical Chemistry, the Institute of Chemical Biology and Fundamental Medicine (ICBFM), Siberian Branch of the Russian Academy of Sciences (SB RAS) (Novosibirsk, Russia). Oligoribonucleotides utilized as siRNAs were synthesized and purified in the RNA chemistry laboratory (ICBFM SB RAS). The list of siRNAs is presented in Table S2. The NGS of RNA was performed in the SB RAS Genomics Core Facility (ICBFM SB RAS). Rabbit polyclonal antibodies to human NSUN2 (#20854-1-AP) and mouse monoclonal antibodies to recombinant human GAPDH (#60004-1-Ig) were from ProteinTech (Rosemont, IL, USA); secondary rabbit or mouse antibodies conjugated to horseradish peroxidase were from Sigma-Aldrich (St. Louis, MO, USA).

### 4.2. In-Cell Cross-Linking

PAR-CLIP was performed using HEK293T cells in 4 biological replicates according to [23] with some modifications. In a typical experiment, HEK293T cells were grown on 15 cm culture dishes on Dulbecco’s modified Eagle’s medium (DMEM) supplemented with 10% fetal bovine serum (FBS) and antibiotic-antimycotic 100× (all Thermo Fischer Scientific, Waltham, MA, USA). At 80% confluence, s^4^U was added up to the concentration of 250 μM. After 6 h, the medium was aspirated, the cells were washed with ice-cold PBS, placed on ice, and UV-irradiated (365 nm, 1000 mJ/cm^2^) in Bio-link (Vilber Loumart, Marne-la-Vallée, France); the cells were not irradiated in the control experiment. After that, the cells were washed again with ice-cold PBS and pelleted by centrifugation at 1000× *g* (1 min, 4 °C), and then the cells (30 × 10^6^ cells for each replicate) were lysed, triturated through a G30 needle, and centrifuged at 20,000× *g* (15 min, 4°C). The lysate was treated with RNase T1 (0.5 U/μL) for 20 min at 25 °C and incubated overnight at 4 °C with the Protein G-conjugated magnetic beads (Dynabeads, Life Technologies, Carlsbad, CA, USA) pre-bound to polyclonal antibodies to NSUN2 (#20854-1-AP, ProteinTech). After immunoprecipitation, the beads were incubated with RNase T1 (2 U/μL), washed, incubated with calf intestinal phosphatase (NEB), and then washed again. RNA fragments cross-linked to NSUN2 were ^32^P-labeled on the beads, washed, and eluted by heating in sodium dodecyl sulfate polyacrylamide gel electrophoresis (SDS-PAGE) sample buffer. The resulting eluate was resolved on 10% SDS-PAGE, after which the gel was blotted onto a nitrocellulose membrane, followed by exposure to a Phosphorimager screen (Bio-Rad, Hercules, CA, USA). The radioactive signals were scanned using a Pharos FX Plus Molecular Imager with QuantityOne software (Bio-Rad). The RNA fragments were isolated as described in [21].

### 4.3. Preparation of cDNA Libraries from Cross-Linked RNA Fragments, NGS, and Bioinformatics Analysis of the PAR-CLIP-Derived Data

Raw data generated by 3 runs (1 on MiSeq (Illumina, San Diego, CA, USA) and 2 on MGIseq 2000 (BGI, Shenzhen, China) for each replicate) were initially processed as described in [37]. The pre-processed fastq files were merged, filtered using trimmomatic (minlen = 13), and mapped to the reference genome using the STAR RNA aligner (v.2.7.1.a). Only uniquely mapped reads were saved after filtering (MAPQ > 10, samtools view–q10). The main analysis was performed with the wavClusteR package (v. 2.28.0) with default parameters (minCov = 10) and using the merged over all replicates BAM file obtained in the previous step as an input. The interval of relative substitution frequencies for high-confident T/C transitions induced by PAR-CLIP was estimated between 0.007 and 0.98. The clusters of reads with T/C-transitions were identified with the BSgenome.Hsapiens.NCBI.GRCh38 package (v. 1.3.1000). The resulting clusters containing high-confident characteristic T/C-transitions were annotated using the hiAnnotator package (v. 1.28.0); ensembl v.104 and gencode.v40.tRNAs databases were used as the annotation sources for the main genomic features and for predicted tRNA genes, respectively. All the PAR-CLIP read data were submitted to GenBank under the study accessions PRJNA853906.

### 4.4. Cell Culture, NSUN2 mRNA Knockdown, and Determination of NSUN2 Content

HEK293T cells were cultured in 10 cm dishes as described above. At 30% confluence, cells were transfected with specific siRNAs against the mRNA of NSUN2 using Lipofectamine 3000 according to the manufacturer’s protocol. After 48 h, the cells were passaged, transfected with NSUN2-specific siRNAs again, and harvested 48 h later at 80–90% confluence. To this end, the cells were placed on ice, washed with ice-cold PBS, kept for 10 min on ice with PBS and 0.1 mg/mL of cycloheximide, resuspended, and pelleted by centrifugation at 500× *g* (30 s, 4 °C). In control experiment, the set of non-targeting siRNAs was used. To confirm the knockdown of the mRNA of NSUN2, the content of the protein itself in the cells was determined by Western blotting using specific rabbit polyclonal antibodies against NSUN2 as described in [38]. Rabbit antibodies against GAPDH were used as a reference.

### 4.5. Preparation of RNA Samples from NSUN2 Knockdown Cells

For NGS, RNA samples in 4 biological replicates from one 10 cm plate of NSUN2 knockdown HEK293T cells were utilized. To isolate the samples for RNA-seq analysis, 1/5 cells from each replicate were lysed with the Trizol reagent. To isolate the samples for polysome profiling followed by RNA-seq analysis, 4/5 of cells from each replicate were lysed on ice for 10 min in 20 mM Tris-HCl (pH 7.5) containing 15 mM MgCl_2_, 200 mM KCl, 1% Triton X100, and 0.1 mg/mL of cycloheximide. The lysates were clarified by centrifugation for 1 min at 14,000× *g* and layered on sucrose-density gradients (7−47%) in 50 mM Tris-HCl (pH 7.5) containing 100 mM KCl and 12 mM MgCl_2_ and centrifuged at 20,500× *g* in Beckman SW40 rotor (16 h, 4 °C). The gradients were then fractionated, and the A_260_ of the fractions was measured. Fractions corresponding to polysomes were supplemented with one volume of EtOH and MgCl_2_ (up to 20 mM) and centrifuged at 14,000× *g* (30 min, 4 °C). Polysome pellets were dissolved in the Trizol reagent. To evaluate the level of NSUN2 knockdown in the cells used, 1/100 of the lysate was analyzed by SDS-PAGE, and then the separated proteins were transferred from the gel onto a nitrocellulose membrane followed by immunoblotting as described above. To assess the ratio of 80S ribosomes in the polysome and monosome fractions (P/M), the integrated A_260_ values of the respective peaks were calculated in the MS Excel program. All other manipulations, including the isolation of RNA from the samples of cells and polysomes lysed with the Trizol reagent, along with the RNA integrity index assessment, RNA quantification, and RNA polyA enrichment, were carried out in accordance with the described protocol [38].

### 4.6. Preparation of DNA Libraries for RNA-Seq, Their NGS, and the Bioinformatics Processing of the Data Obtained

The quality-checking of RNA samples, DNA libraries preparation, their high-throughput sequencing with the use of the MGISEQ-2000 platform (MGI Tech, Shenzhen, China), and raw data processing were performed as described in [34]. The resulting BAM files were indexed, and their quality was checked using the QualimapTool (v.2.2). All the RNA-seq read data were submitted to GenBank under the study accessions PRJNA853906. The baseMean values obtained from the RNA-seq experiment with the total mRNA of HEK293T cells were used to plot the mRNA abundance in MS Excel. Differential expression analysis (DEA) was carried out using the DESeq2 (v. 1.30.0) package in general, as described in [39]. Raw counts were estimated using the Rsubread package; annotation was performed using the biomaRt package. The PCA plots were generated using the *plotPCA* function from the DESeq2 package based on rlog transformed read counts as described in the DESeq2 manual. DESeq2 with default parameters was utilized to perform DEA with the RNA-seq data and the polysome profiling followed by RNA-seq data separately, as well as when determining GATEs using the algorithm described in the systemPipeR package vignette. For the selection of DEGs, the *p* value adjusted (*p*.adj) cut-off was assigned to <0.05, the absolute shrunken LFC cut-off was assigned to >0.585, and the baseMean value was assigned to higher than 100, which excludes poorly covered genes, i.e., weakly expressed ones. Volcano plots were generated using the EnhancedVolcano package (v. 1.4.0). Calculations of translational efficiencies of genes were performed with the DESeq2 package. The sequences of mRNAs were extracted using the Biostrings package. The GO enrichment analysis of DEGs against the GO terms was carried out using the online-based resource www.geneontology.org (accessed on 15 May 2022) by selecting the Fisher’s Exact test type and False Discovery Rate (FDR) correction parameters. The motif discovery was carried out using MEME Suite. Secondary structure analysis was performed with the RNAFold tool from ViennaPackage (v.2.5.1). Motif analysis was carried out with the Streme tool from MEME Suite (v. 5.4.0), and the sequences of mRNA parts of non-DEGs were used as control.

### 4.7. Validation of RNA-Seq and Poly-Seq Data by RT-qPCR

For reverse-transcription, 2 g of total RNA from HEK293T cells with and without the knockdown of NSUN2 or from the respective polysome fractions was incubated with 100 pmol of random primer and 25 U of MMLV reverse-transcriptase according to the protocol (Thermo Fischer Scientific, Waltham, MA, USA). qPCR was performed on the LightCycler 96 (Roche) using the SYTO9 fluorescent dye (Thermo Fischer Scientific), HS-Taq polymerase (Biolabmix, Russia), and gene-specific primers. The experiments were carried out for each of 3 biological replicates in 2–3 technical ones. The reaction conditions were as follows: 95 °C for 30 s; 45 cycles 95 °C for 10 s, 57 °C for 20 s, and 72 °C for 20 s (single acquisition). The relative quantification of gene expression levels was determined using R package *pcr* [40]. The reference genes were those encoding GAPDH and 18S rRNA.

## 5. Conclusions

The effects of a deficiency of the RNA cytosine C5 methyltransferase NSUN2 on the composition of the transcriptome and translatome in mammalian cells enabled determining the dependence of gene expression on the methylation of mRNAs. A decrease in the NSUN2 level enhances the efficiency of translation of a large group of specific mRNAs, which includes shorter ones with a higher GC content, where motifs with C residues are located in GC-rich environments. The PAR-CLIP assay applied to endogenous NSUN2 revealed a group of its target mRNAs and its binding sites in them, showing the preferential location of these sites predominantly in the CDSs in GC-rich regions with a pronounced secondary structure. A fairly significant overlap between the sets of mRNAs that are targets of YB-1, the main packager of mRNAs that is most probably involved in their exosomal export, and of NSUN2 suggests that the latter may be implicated in the regulation of mRNA sorting into exosomes. The next research frontier in this area may be the discovery of a mechanism by which NSUN2 affects the cellular pathways related to the packaging of mRNAs, their sorting into exosomes, and extracellular export.

## Data Availability

The PAR-CLIP and RNA-seq read data reported in this study were submitted to GenBank under the study accession PRJNA738595.

## Supplementary Materials

The following supporting information can be downloaded at: https://www.mdpi.com/article/10.3390/ijms23179740/s1.
