# Supplementary material for: Reorganization of the Landscape of Translated mRNAs in NSUN2-Deficient Cells and Specific Features of NSUN2 Target mRNAs
Source: Int J Mol Sci. 2022 Aug 28;23(17):9740. doi: 10.3390/ijms23179740 (PMC9456143; doi:10.3390/ijms23179740)
Supplement: Supplementary file 1 [file ijms-23-09740-s001.zip › ijms-1840233-supplementary.pdf]

Supplementary Materials

Figures

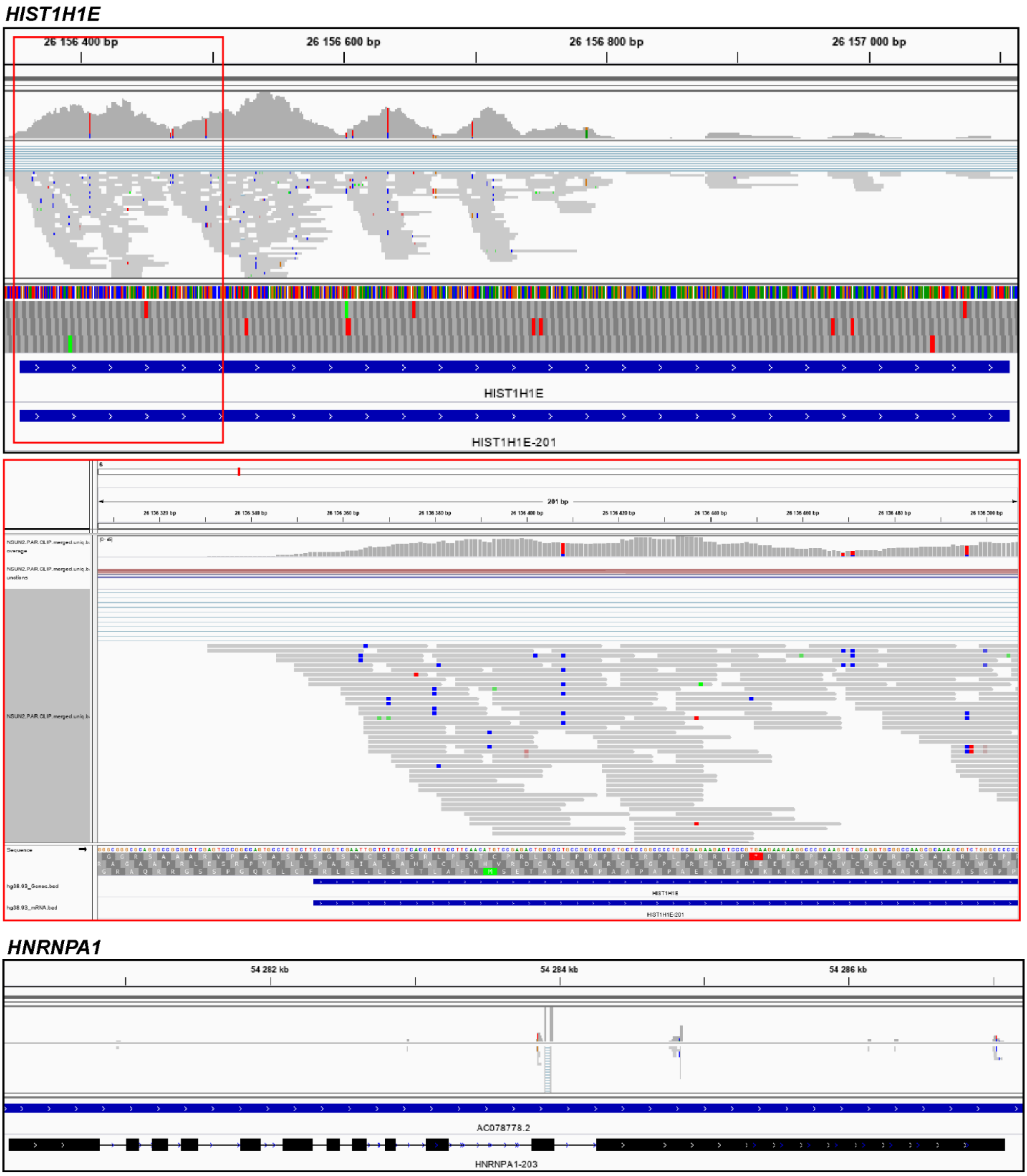

Figure S1. The IGV genome browser view of regions of the human genome corresponding to the *HIST1H1E* gene with the mapped sequencing reads obtained by the NGS of the RNA fragments cross-linked to NSUN2. Red box shows zoomed region of the browser view. The positions of the T/C transitions in the reads are visible as vertically repeating blue dashes above the T letters in the sequence line. View of the *HNRNPA1* gene regions showing no reads is presented as a control for the specificity of pull-down of the NSUN2-cross-linked mRNA fragments.

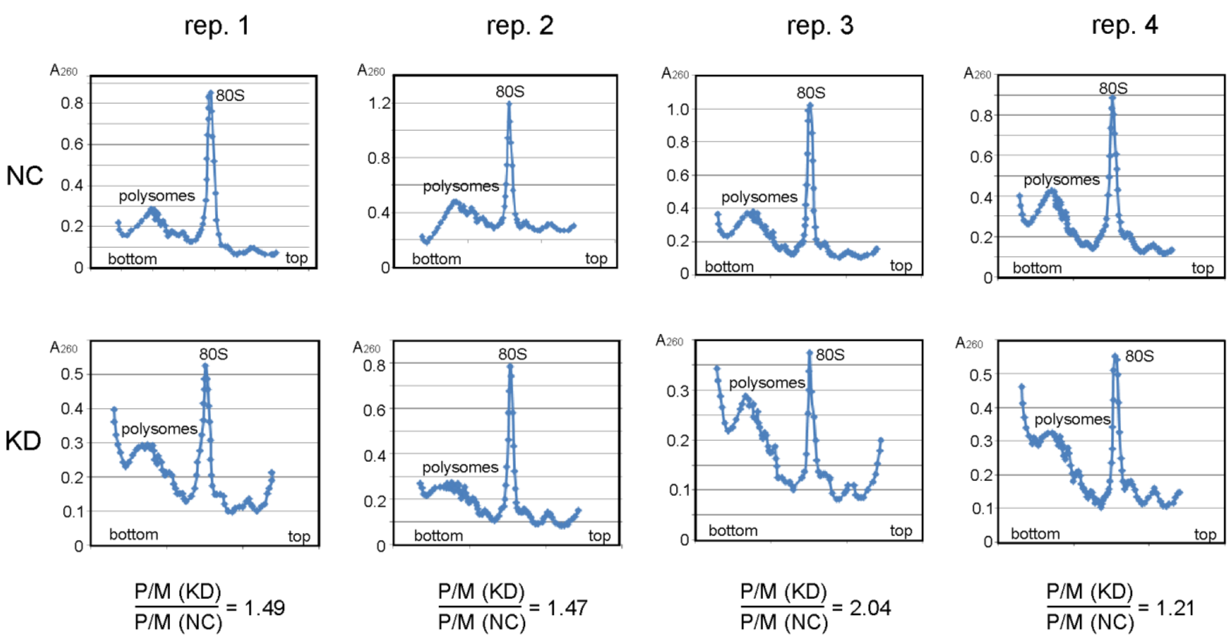

Figure S2. Polysome profiles obtained by centrifugation in sucrose-density gradient of the lysates from HEK293T cells transfected with either NSUN2 mRNA-specific siRNAs (KD) or non-targeting siRNAs (NC) in four replicates. Peaks corresponding to polysomes and 80S monosomes are marked. The ratios P/M(KD):P/M(NC) determined from replicates are shown.

Tables

Table S1. The metadata of NSUN2 cellular RNA-targets identified by PAR-CLIP (the Excel table). (Sheet 1) The comprehensively annotated clusters of reads containing T/C-transitions identified with the use of the wavClusteR. (Sheet 2) The distribution of the read clusters across mRNA features. (Sheet 3) The annotated clusters containing T/C-transitions in protein-coding genes. (Sheet 4) A comparison of RNA-targets for NSUN2 (this work) and YBX1 (ref. [21] in the main text) identified by PAR-CLIP.

Table S2. The sequences of siRNAs used in this work. The sense (S) and antisense (AS) strands of siRNA duplexes targeting to NSUN2 mRNA (NSUN2) or of those non-targeting to it, control (NC), are shown.

Index	Sequence
NSUN2-S1	5’-ggaugguguauuccacgug**dtdt**-3’
NSUN2-AS1	5’-cacguggaauacaccauccc**dtdt**-3’
NSUN2-S2	5’-cacguguucacuaaacccuau**dtdt**-3’
NSUN2-AS2	5’-auaggguuuagugaacacgug**dtdt**-3’
NC-S1	5’-uucuccgaacgugucacgu**dtdt**-3’
NC-AS1	5’-acgugacacguucggagaa**dtdt**-3’
NC-S2	5’-uuguucgaacgugucacgu**dtdt**-3’
NC-AS2	5’-acgugacacguucgaacaa**dtdt**-3’

Table S3. The differential expression analysis results of the RNA-seq data obtained with total RNA from cells with and without NSUN2 knockdown (Excel table). (Sheet 1) The metadata obtained with the use of DESeq2. (Sheets 2 and 3) The subsets of tDEGs with cutoffs (p adj < 0.05, ǀLFCǀ > 0.585).

Table S4. The results of the differential expression analyses of the RNA-seq data obtained with polysome-associated mRNA from cells with and without NSUN2 knockdown (Excel table). (Sheet 1) The

metadata obtained with the use of DESeq2. (Sheets 2 and 3) The subsets of pDEGs with cutoffs (p adj < 0.05, ∣LFC∣ > 0.585).

Table S5. The GO enrichment analysis (Excel table). Analyses of the sets of up-regulated (up) and down-regulated (down) tDEGs, pDEGs and GATEs in the biological process (BP) and cellular component (CC) categories.

Table S6. The analysis of genes with altered translational efficiencies (GATEs) based on the NGS data obtained with total and polysome-associated mRNA from cells with and without NSUN2 knockdown (Excel table). (Sheet 1) The metadata obtained with the use of DESeq2. (Sheets 2 and 3) The subsets of up-regulated (up) and down-regulated (down) GATEs with cutoffs (p adj < 0.05, ∣LFC∣ > 0.585).

Table S7. The list of oligonucleotide primers used for RT-qPCR.

Gene	Forward primer	Reverse primer
NSUN2	5′-gaacttgcctggcacacaaat-3′	5′-tgctaacagcttcttgacgacta-3′
TPM4	5’-aatttgcagagagaacggttgc-3’	5’-cagtgtctgatgtaagcccac-3'
SRM	5’-ctaccaggacatcctcgtcttc-3’	5’-agaggcaggttggcgatcatct-3’
NAMPT	5’-atcctgttccaggctattctgt-3'	5’-ccccatattttctcacacgcat-3'
SRP54	5’-aacttcttggtatgggcgaca-3’	5’-gtctcgcaacgtaaactgacc-3’
SEPTIN7	5'-ctcacaccagaggaatgccaac-3'	5’-ccacagcaagaggtaaacggtc-3’
STMN3	5'-tgctcctgcttctacacacagc-3'	5'-gacaggtcagaaggggacttga-3'
GAPDH	5’-gtgaaccatgagaagtatgacaac -3’	5’-catgagtccttccacgatacc -3’
